# The Study of Medical Ethics

**Published:** 1861-03

**Authors:** 


The Study of Medical Ethics.—The Code of Ethics of the American Medical Association has now been the recognized standard of medical morals in this country for nearly fourteen years. It was prepared by the wisest members of our profession, among whom we recognize the honored and trustworthy names of Drs. Bell, Hays, and Emerson, of Philadelphia; Prof. Clark, of N. Y, and Prof. Arnold, of Ga. When submitted to the Convention of 1847, the Code was adopted unanimously. Since that period no one has dissented from its provisions, but every legitimate medical organization in the country has adopted it; and thus it stands as our organic medical law. This document defines with admirable simplicity and purity of language, and with the nicest appreciation of the exalted spirit of scientific medicine, the duties of physicians to each other as members of a liberal profession, and the reciprocal obligations which exist between them and the individual members of society. It is, in a word, the guide to the formation of a true medical character. And yet how little is this regarded by physicians, and how few are familiar with its admirable provisions! Of the hundreds of graduates who are annually introduced to the ranks of the profession, how few are aware of even the existence of such a chart to professional excellence, much less imbued with its spirit!

There are at this time between four and five thousand medical students receiving instruction at the various colleges in this country. These young gentlemen are daily and sufficiently drilled in anatomy, physiology, chemistry, microscopy, obstetrics, and therapeutics, while they are employed far into the night in dissecting, and thus verifying upon the dead subject the text of the morning lecture. Class after class thus entering college, is graduated with honors more or less emphatic, and joins the great procession of hygeian ministers in the world at large. It is usual, on the commencement day, for some venerable physician to address the departing graduates on the well known duties and responsibilities awaiting them in their new relations to society; to encourage them by the hope of success; to stimulate their ambition by the examples of great lives which have adorned the profession; then, with a parting blessing, the young Esculapians are dismissed to their great encounter with the realities of medical practice.

Now, except in the commencement address, where acciden tally it may be alluded to, we would ask whether it is usual in any of our medical schools to deliver any *set* lectures on medical ethics? Is even *one* annually and invariably delivered to the students? We ask this for information, because we have never heard that any such dissertations were read as part of the curriculum of instruction.

Admitting this to be so, the inquiry naturally arises whether our colleges can be said to do their whole duty towards students in fitting them to practice successfully, when they fail to instruct them in those rules of professional intercourse whose observance brings them, antecedently even to intellectual merits, the approbation of their fellow-practitioners, and on the contrary, whose violation insures them the certain and immediate reprobation and scorn of their professional brethren. If an individual wishes to rise to meritorious eminence in any profession, he must, first of all things, secure to himself the sympathy and the respect of his fellow-laborers. Without that he can never permanently sustain his status among gentlemen. For, although he may rise spasmodically, and flutter in mid air awhile upon waxen wings, yet the inexorable sunlight of Truth will speedily dissolve these frail supports, and leave him to flounder among the shoals of pretenders who swarm in the lower depths of the profession.

It does not follow because a man's brain is as full of learning as Lord Bacon's, that he may not at the same time be a most unmitigated boor, whose self-conceit or selfishness leads him alike to trample upon the rights and the feelings of his professional brethren, in his insensate haste to become rich, or to gain the bubble reputation. These things are of too frequent occurrence not to have been noticed by all, and it is not difficult in any community to point out some physicians who, great enough in intellectuality, are yet moral idiots in respect to the dignity and the honor of the profession they follow. Such men, whatever their talents, their wealth, or their factitious distinctions, are still living in virtual outlawry to the canons of medical ethics, nor can the ephemeral praises of an indiscriminate press indemnify them for the lost sympathy and respect of their fellows. Pitiable indeed is the condition of that man who is shunned by his peers, whose name provokes only contempt, and who is dismissed from the thoughts as one fallen from the high estate of a Christian gentleman and an honorable man.

Let these things, in all their length, breadth and strength of application, be taught to the young men in our medical colleges. Let them understand that the moral side of a physician's character is quite as important as the intellectual. Nay, that in advance even of any knowledge of his intellectual capacities, the public will be favorably inclined towards him whom his fellow practitioners recommend and advance. It would take no large amount of time, nor make any serious interruption in the course of medical studies, to have one lecture a week delivered on the subject of professional ethics. There are gentlemen enough in and out of our medical faculties who would be happy to do thus much to preserve the dignity of the profession; who would be willing to instruct students in that code of medical ethics which is the basis of professional respectability. And, in particular, it will be a source of satisfaction and pride to our colleges to know that, besides making physicians, they have made men of refinement and dignity. Each faculty in its own college thus becomes a *humanizer*, as well as an educator of young men.

In this fervent hope we now commend the subject to the earnest attention of our medical schools, not doubting that they will see in these crude suggestions the inklings of so much truth as will prompt them to incorporate in the course of their instruction some few lectures on medical ethics.—*American Medical Times.*

